# Patients with high serum substance P levels previously to liver transplantation for hepatocellular carcinoma have higher risk of one-year liver transplantation mortality

**DOI:** 10.18632/oncotarget.25097

**Published:** 2018-04-20

**Authors:** Leonardo Lorente, Sergio T. Rodriguez, Pablo Sanz, Antonia Pérez-Cejas, Javier Padilla, Dácil Díaz, Antonio González, María M. Martín, Alejandro Jiménez, Purificación Cerro, Manuel A. Barrera

**Affiliations:** ^1^ Intensive Care Unit, Hospital Universitario de Canarias, Santa Cruz de Tenerife, 38320, Spain; ^2^ Intensive Care Unit, Hospital Universitario Nuestra Señora Candelaria, Santa Cruz de Tenerife, 38010, Spain; ^3^ Department of Surgery, Hospital Universitario Nuestra Señora de Candelaria, Santa Cruz de Tenerife, 38010, Spain; ^4^ Laboratory Department, Hospital Universitario de Canarias, San Cristóbal de La Laguna, 38320, Spain; ^5^ Department of Digestive, Hospital Universitario Nuestra Señora de Candelaria, Santa Cruz de Tenerife, 38010, Spain; ^6^ Research Unit, Hospital Universitario de Canarias, San Cristóbal de La Laguna, 38320, Spain; ^7^ Transplant Unit, Hospital Universitario Nuestra Señora Candelaria, Santa Cruz de Tenerife, 38010, Spain

**Keywords:** substance P, hepatocellular carcinoma, liver transplantation, mortality, outcome

## Abstract

**Purpose:**

Substance P is a tachykinins family member with inflammatory effects. Higher circulating levels of substance P have been found in patients with liver diseases and in patients with higher severity of liver diseases. The objective of this study was to determine whether serum levels of substance P levels, prior to liver transplantation (LT) for hepatocellular carcinoma (HCC) are associated with one-year LT mortality.

**Material and Methods:**

In this observational retrospective unicenter study were included patients with LT for HCC. Serum levels of substance P were measured before LT. The end-point of the study was one-year mortality after LT.

**Results:**

We found that one-year survivor patients (*n* = 127) showed a lower age in liver donors (*p* = 0.03) and lower levels of serum substance P levels (*p* = 0.003) than non-survivor patients (*n* = 15). Logistic regression analysis showed that serum levels of substance P (levels) were associated with one-year mortality (Odds Ratio = 1.011; 95% CI = 1.004–1.018; *p* = 0.002) controlling for the age of the LT donor.

**Conclusions:**

We believe that our study is the first study reporting data on circulating levels of substance P previously to LT for HCC, and an association between elevated levels of serum substance P before LT and mortality during the first year of LT.

## INTRODUCTION

Hepatocellular carcinoma (HCC) is the most frequent primary hepatic malignant tumor, and is the second cause of death attributable to cancer. Liver transplantation (LT), which eliminate the hepatic tumor and treats the hepatic insufficiency, is the treatment of choice in some patients with HCC [1–7].

The Tachykinin family has different members as neurokinin A (NKA), neurokinin B (NKB), substance P, and endokinins [8–16]. There are three tachykinin receptors (NK1R, NK2R, and NK3R). NKA has a preferential binding with NK2R, NKB with NK3R, and SP and endokinins with NK1R. Tachykinins are distributed by central and peripheral nervous systems, respiratory, urinary and immune systems, gut and blood vessels. Tachykinins are involved in the transmission of nociceptive responses, airway contraction, inflammation, vasodilation, plasma protein extravasation, smooth muscle contraction, and salivary secretion. Substance P is implicated in central and peripheral nervous systems injury, inflammatory bowel disease, psoriasis, migraine, and asthma [8–16].

Elevated circulating levels of substance P have been found in patients with liver diseases than in control subjects [17–23], and in patients with higher severity of liver diseases [21–23]. However, there is not published data about circulating levels of substance P in patients with HCC underwent to LT. Consequently, the objective of this study was to determine whether serum levels of substance P previously to LT for HCC are associated with one-year LT mortality.

## MATERIALS AND METHODS

### Design and subjects

In this observational retrospective unicenter study were included patients with LT for HCC in the Hospital Universitario Nuestra Señora de Candelaria (Santa Cruz de Tenerife, Spain) from January 1996 to January 2016. LT proceeded in all cases from brain death donor. The study was carried out by the approval of Institutional Review Board, and the written informed consent from the patients or their family members.

Previously, we determined serum levels of malondialdehyde [24], caspase-cleaved cytokeratin-18 [25], and sCD40L [26] in some of those patients. In the current work, serum levels of substance P were analyzed to determine their association with LT mortality at one year.

### Variables recorded

Child-Pugh score [27], inside Milan criteria [28] previously and after LT, model for end-stage liver disease (MELD) score [29] by hepatic function, degree of tumor differentiation, age of liver receptor, infiltration, age of liver donor, macrovascular invasion, microvascular invasion, portal hypertension (determined clinically or by hepatic venous pressure gradient), LT technique, multinodular tumor, treatment previously to LT, serum alpha-fetoprotein (AFP) levels, size nodules ABO blood type, and sex were recorded for all patients. One-year survival was the end-point of the study.

### Determination of serum levels of substance P

Serum blood samples were obtained before LT, and serum levels of substance P were determined at Laboratory Department of Hospital Universitario de Canarias (La Laguna, Spain). We used a competitive enzyme immunoassay designed to measure substance P in serum and other biological fluids (R&D Systems, Abingdon, UK). This immonoassay contains a synthetically derived human substance P peptide and has been shown to accurately quantitate this peptide. This kit can be used to determinate relative mass values for natural substance P. The assay was performed on an automatic analyser, the EVOLIS™ System, a self contained micoroplate processor for automated enzyme immunoassay testing.Substance P kit has an intra-assay coefficient of variation (CV) of 9%, an inter-assay CV of 15%, and a detection limit of 25 pg/ml.

### Statistical methods

Frequencies and percentages were used to report categorical variables (compared between groups by chi-square test), and medians and interquartile ranges to report continuous variables (compared between groups by Mann–Whitney test). A receiver operating characteristic (ROC) curve was carried out to determine the one-year mortality prognostic capacity of serum levels of substance P. A Kaplan-Meier survival analysis was performed using one-year LT survival (dependent variable), and serum levels of substance P lower/higher than 182 pg/mL (independent variable); and this cut-off of serum levels of substance P was selected in basis to Youden J index. A logistic regression analysis was carried out to determine the association between serum levels of substance P and one-year mortality of LT controlling for LT donor age; and the clinical impact of the predictor variables was estimated by Odds Ratio and its 95% confidence intervals (CI). Statistical analyses were performed by SPSS 17.0 (SPSS Inc., Chicago, IL, USA) and MedCal 15.2.1 (Ostend, Belgium), and *P*-values < 0.05 were considered statistically significant.

## RESULTS

We included 142 patients in the study. Of them, 21 (14.8%) were females, 136 (95.8%) were inside Milan criteria previously to LT and 117 (82.4%) after LT, 98 (69.0%) had portal hypertension, 44 (30.9%) multinodular tumor, 44 (30.9%) infiltration, 7 (5.9%) macrovascular invasion, and 30 (21.1%) microvascular invasion. A total of 15 (10.6%) patients dead during the first year of LT and 127 (89.4%) were lives.

It was found that the surviving patients of one-year (*n* = 127) showed a lower age in liver donors (*p* = 0.03) and lower levels of serum substance P (*p* = 0.003) than the non-survivor patients (*n* = 15) (Table 1). However, Child-Pugh score, inside Milan criteria previously and after LT, MELD score, degree of tumor differentiation, age of liver receptor, infiltration, macrovascular invasion, microvascular invasion, portal hypertension, LT technique, multinodular tumor, treatment previously to LT, serum levels of AFP, size nodules ABO blood type, and sex were not statistically significant differences between one-year survivor and non-survivor patients (Table 1).

**Table 1:** Clinical characteristics of one-year liver transplantation survivor and non-survivor patients

	1 year survivors patients (*n* = 127)	1 year non-survivor patients (*n* = 15)	*p*
Child-Pugh score–*n* (%)			0.42
·A	62 (48.8)	10 (66.7)	
·B	36 (28.3)	3 (20.0)	
·C	29 (22.8)	2 (13.3)	
ABO blood type–*n* (%)			0.91
·A	59 (46.5)	6 (40.0)	
·B	11 (8.7)	2 (13.3)	
·O	51 (40.2)	6 (40.0)	
·AB	6 (4.7)	1 (6.7)	
Gender female–*n* (%)	21 (16.5)	0	0.13
Inside Milan criteria previously to LT–*n* (%)	122 (96.1)	14 (93.3)	0.50
Inside Milan criteria after LT–*n* (%)	106 (83.5)	11 (73.3)	0.30
Degree of tumor differentiation–*n* (%)			0.48
·Well	95 (74.8)	12 (80.0)	
·Moderate	29 (22.8)	2 (13.3)	
·Poor	3 (2.4)	1 (6.7)	
Multinodular tumor–*n* (%)	39 (31.5)	5 (33.3)	0.99
Portal hypertension–*n* (%)	87 (65.8)	11 (73.3)	0.99
Infiltration–*n* (%)	40 (31.5)	4 (26.7)	0.99
Macrovascular invasion–*n* (%)	7 (5.5)	0	0.99
Microvascular invasion–*n* (%)	27 (21.3)	3 (20.0)	0.99
Transplantation technique–*n* (%)			0.78
·By-pass	44 (34.6)	6 (40.0)	
·Piggy back	83 (65.4)	9 (60.0)	
Treatment previously to LT–*n* (%)	69 (54.3)	10 (66.7)	0.42
·Percutaneous ethanol injection (PEI)–*n* (%)	28 (22.0)	7 (46.7)	0.054
·Radiofrequency ablation (RFA)–*n* (%)	8 (6.3)	0	0.99
·Transarterial chemoembolization (TACE)–*n* (%)	27 (21.3)	3 (20.0)	0.99
·Liver resection–*n* (%)	3 (2.4)	0	0.99
·Mixed treatment–n (%)	3 (2.4)	0	0.99
MELD score - median (p 25–75)	15 (12–18)	15 (15–18)	0.85
Nodules size (cm) - median (p 25–75)	3.0 (2.0–3.5)	3.2 (1.7–4.6)	0.70
Age of LT recipient (years) - median (p 25–75)	59 (52–62)	56 (53–62)	0.92
Leukocytes count–median × 10^3^/mm^3^ (p 25–75)	4.90 (3.60–6.25)	4.94 (3.49–7.92)	0.67
Albumin (g/dL) - median (p 25–75)	3.33 (2.90–4.10)	3.31 (2.93–4.16)	0.95
Protein (g/dL) - median (p 25–75)	6.70 (6.10–7.10)	6.70 (5.70–7.68)	0.86
Age of liver donor (years) - median (p 25–75)	52 (35–63)	62 (49–72)	0.03
Serum substance P (pg/mL)–median (p 25–75)	138 (81–210)	229 (144–331)	0.003

LT: liver transplantation; MELD: model for end-stage liver disease.

Logistic regression analysis showed that serum levels of substance P higher than 182 pg/mL were associated with one-year mortality (Odds Ratio = 5.773; 95% CI = 1.681–19.828; *p* = 0.005) controlling for the age of the LT donor (Table 2).

**Table 2:** Logistic regression analysis for the variables associated with one-year liver transplantation mortality

	Odds Ratio	95% Confidence Interval	*p*-value
Serum substance P levels > 182 pg/mL	5.773	1.681–19.828	0.005
Age of liver donor (age)	1.050	1.002–1.100	0.04

**Figure 1:**
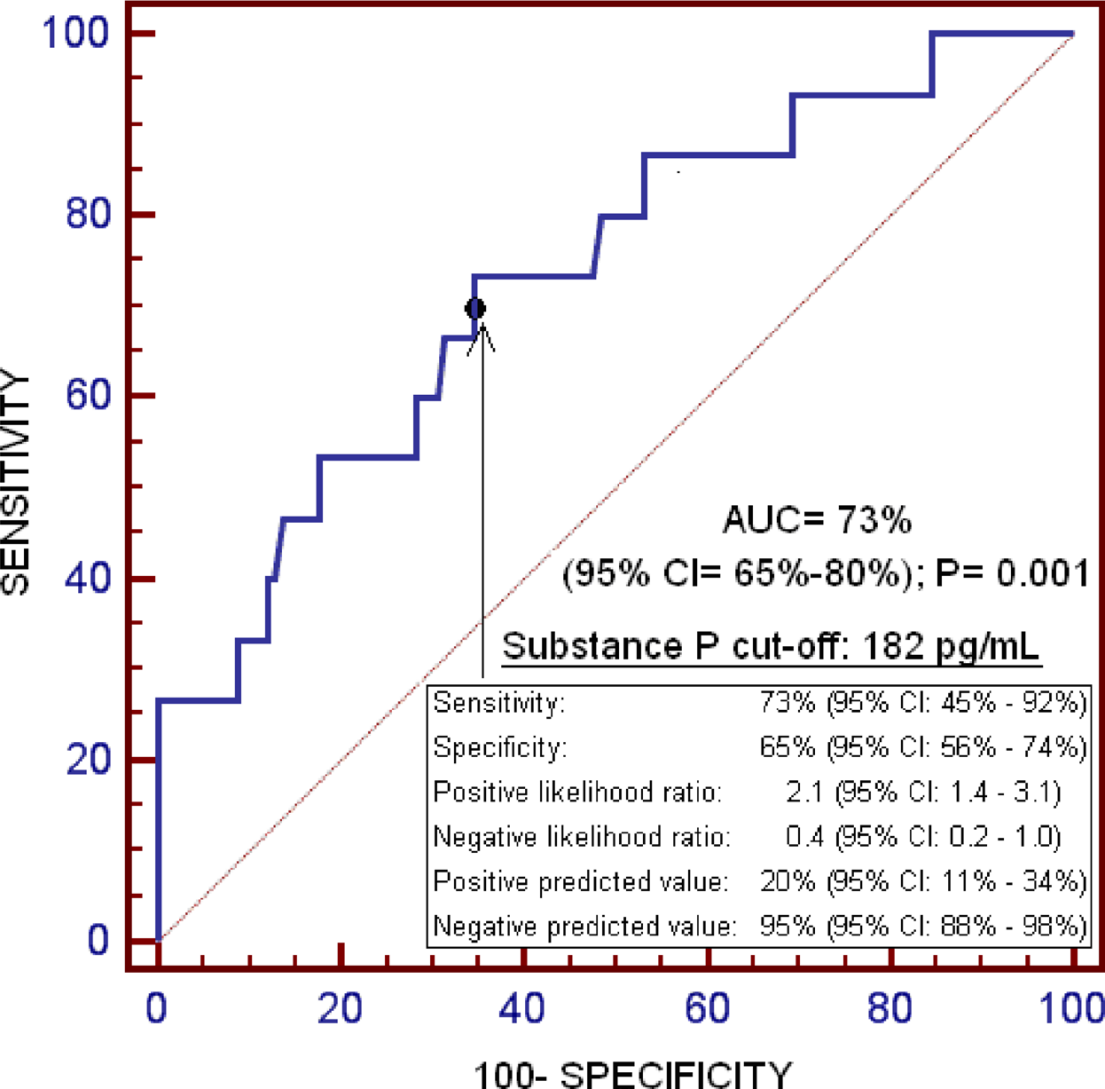
Receiver operation characteristic of serum substance P levels for one-year liver transplantation mortality prediction

ROC analysis showed that the area under the curve of serum levels of substance P to predict one-year LT mortality was of 73% (95% CI = 65%–80%; *p* = 0.001) (Figure 1).

**Figure 2:**
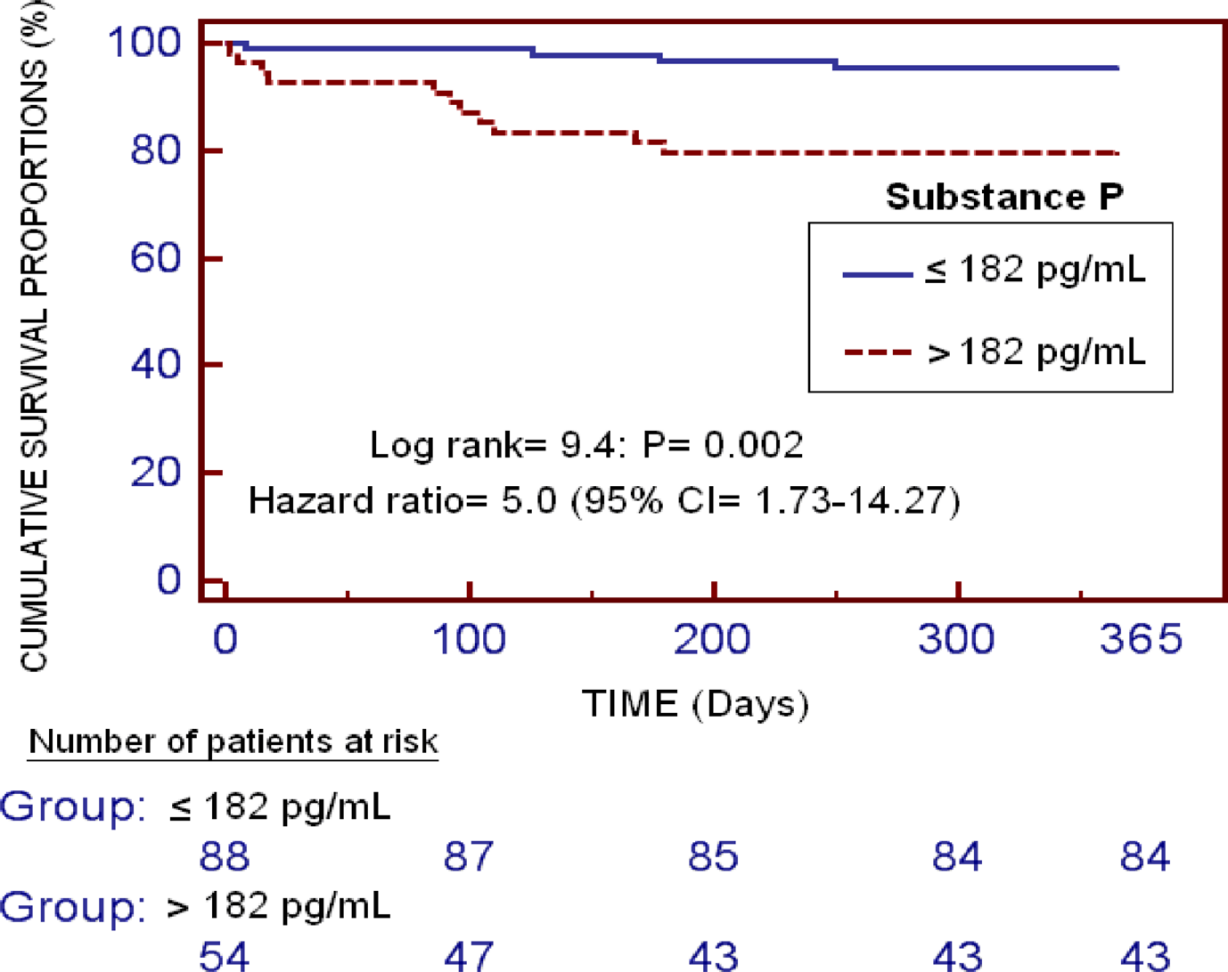
Kaplan-Meier survival analysis using one-year LT (dependent variable) and serum substance P levels lower/higher than 182 pg/mL (independent variable)

Kaplan–Meier survival analysis showed that patients with serum levels of substance P higher than 182 pg/mL had a higher risk of death during the first year of LT (Hazard Ratio = 9.4; 95% CI = 1.73–14.27; *p* = 0.003) than patients with lower concentrations (Figure 2).

## DISCUSSION

We believe that our study is the first study reporting data on circulating levels of substance P previously to LT for HCC, an association between elevated levels of serum substance P before LT and mortality during the first year after LT controlling for liver donor age, and that serum levels of substance P before LT could be used for the prognosis estimation of one-year survival after LT.

Previously, higher circulating levels of substance P have been found in patients with liver diseases than in healthy controls [17–23], and in patients with higher severity of liver diseases [21–23]. In one study of cirrhotic patients, plasma levels of substance P were higher in decompensated patients than in compensated patients, and directly correlated with Child-Pugh's score [21]. Another study with cirrhotic patients found that plasma levels of substance P directly correlated with Child-Pugh's score and inversely correlated with urinary sodium excretion [22]. In another study of patients with hepatic coma was found that plasma levels of substance P inversely correlated with systemic vascular resistance and directly correlated with cardiac index, and that plasma levels of substance P were higher in those patients who finally died in coma [23]. Thus, the association between elevated serum levels of substance P previously to LT and mortality during the first year after LT is a new finding of this study. In addition, the findings of this investigation are in consonance with those found in other pathologies due to that elevated serum levels of substance P have been associated with higher mortality in patients with brain trauma injury [30] and ischemic stroke [31].

Substance P participates in inflammatory response due to that is involved in the production of inflammatory cytokines as interleukin (IL)-1, IL-6 and tumor necrosis factor-α [32–36], prostaglandins [37, 38], nitric oxide [39], and reactive oxygen species (ROS) [40, 41]. It is possible, that non-survivor LT patients remains with higher serum levels of substance P than survivor patients during the first year after LT.

The use of different agents to reduce the activity of substance P has been proven in different pathologies and have been associated with beneficial effects. In animal models of ischemic stroke, the administration of different NK1R antagonists reduced the formation of cerebral edema, permeability of the blood-brain barrier, infarct volume, and functional deficits [42–44]. The use of NK1R antagonists has reduced brain edema and has improved functional outcome in traumatic brain injury animal models [45, 46]. A review examined randomized controlled trials evaluating the effect of tachykinins receptors antagonist agents in patients with asthma, and found that these agents decreased the responsiveness of the airways and improved lung function [47]. Therefore, from a therapeutic perspective, the use of substance P antagonist agents in patients with LT for HCC could be explored to increase their survival.

We recognize some limitations of this investigation. First, there are no data on the circulating levels of inflammatory cytokines. Second, no serum samples were obtained to determine levels of circulating substance P during follow-up. Third, the sample size was not calculated and the non-survival group is relatively small. However was enough large to show an association between serum levels of substance P previously to LT for HCC and one-year liver transplantation mortality controlling for the age of LT donor. Non-survival patient group was relatively small to include other potential variables in the regression model; nevertheless, we included all variables with *p*-value lower than 0.10 in the comparison between non-survivor and survivor patient groups.

## CONCLUSIONS

We believe that our study is the first study reporting data on circulating levels of substance P previously to LT for HCC, an association between elevated levels of serum substance P before LT and mortality during the first year of LT controlling for the age of liver donor, and that serum substance P levels before LT could be used for the one-year survival prognosis estimation.

## Abbreviations

AFP: alpha-fetoprotein
AUC: area under curve
HCC: hepatocellular carcinoma
LT: liver transplantation
MELD: model for end-stage liver disease
